# An Objective Histopathological Scoring System for Placental Pathology in Pre-Eclampsia and Eclampsia

**DOI:** 10.7759/cureus.11104

**Published:** 2020-10-23

**Authors:** Deepak Donthi, Preeti Malik, Anas Mohamed, Aisha Kousar, Ramaswamy Anikode Subramanian, Udaya K Manikyam

**Affiliations:** 1 Pathology, Vidant Medical Center/East Carolina University, Greenville, USA; 2 Public Health, Icahn School of Medicine at Mount Sinai, New York, USA; 3 Pathology, PES Institute of Medical Sciences and Research, Kuppam, IND

**Keywords:** placenta, pre-eclampsia, eclampsia, histopathology, syncytial knots, infarction, villous basement membrane, fibrin deposition

## Abstract

Background and objective

Pre-eclampsia and eclampsia are common complications in pregnancy, and they lead to uteroplacental vascular insufficiency. More than 38% of pregnant women succumb to seizures without meeting the clinical criteria for pre-eclampsia or eclampsia. This highlights the importance of a confirmatory diagnosis of pre-eclampsia or eclampsia using the histopathological changes seen in the placenta. Hence, the present study aimed to validate an objective histopathological scoring system of the placenta for an appropriate diagnosis of pre-eclampsia or eclampsia.

Material and methods

In this prospective study spanning two years, 50 cases of pre-eclampsia/eclampsia and 50 control subjects with normal placenta were included. The histomorphological changes in the placenta were examined for both groups and a scoring system was formulated to assess the severity of pre-eclampsia/eclampsia syndrome. A maximum score of 2 and a minimum score of 0 was assigned for maternal floor infarcts, calcification, villous basement membrane thickening, and fibrin deposition. Syncytial knots were assigned a minimum score of 0 and a maximum score of 1. The association of various placental histopathological variables with a clinical diagnosis of pre-eclampsia, eclampsia, and control was analyzed using the chi-squared/Fisher’s exact test. A one-way analysis of variance (ANOVA) test was used for comparing objective histopathological scores between pre-eclampsia, eclampsia, and control groups. A p-value of less than 0.05 was considered to be statistically significant.

Results

We found a significant association between each histopathological parameters of the placenta, including fibrin deposition, maternal floor infarction, calcification, villous basement membrane thickening, and syncytial knots, and clinical diagnosis of pre-eclampsia, eclampsia, and control groups. A median score of 2 significantly correlated with the normal group, while median scores of 4 and 6 correlated with pre-eclampsia and eclampsia respectively.

Conclusion

This comprehensive scoring system can be a basis for validating reporting patterns of the placenta in pre-eclampsia and eclampsia patients, as well as other disorders related to maternal uteroplacental insufficiency.

## Introduction

Hypertensive disorders of pregnancy, especially pre-eclampsia and eclampsia, are the leading causes of maternal and fetal mortality and morbidity worldwide, accounting for 16% of direct maternal deaths [1]. According to the Global Burden of Disease 2000 report, the pooled incidence of pre-eclampsia in developing countries was estimated to be 3.4%, and it has certainly been on the rise as the years have progressed [2].

In 2014, the definition of pre-eclampsia was revised as hypertension developing after 20 weeks of gestation with one or more of the following conditions: proteinuria, maternal organ dysfunction, or fetal growth restriction [3]. However, high interobserver variability in clinical definition, measurement bias, and errors in assessing hypertension and proteinuria can offer a challenge in diagnosing pre-eclampsia and eclampsia with certainty, as discussed in many previous studies [4-8]. A study by Douglas and Redman reported that 30% of women who presented with eclampsia seizures did not have hypertension and proteinuria, indicating that severe maternal adverse events can occur even in the absence of symptoms that meet the clinical diagnostic criteria of pre-eclampsia [9]. In such scenarios, clinical definitions become obsolete, thereby necessitating the need to confirm such diagnosis by other modalities. According to a prospective cohort study conducted in Sweden, pre-eclampsia in the first pregnancy is a strong predictor of recurrence of pre-eclampsia in future gestations, with an incidence rate as high as 14.7% in the second pregnancy and 31.9% in the third pregnancy [10]. Therefore, confirmed early diagnosis of pre-eclampsia can be helpful in anticipating pre-eclampsia in successive pregnancies.

Furthermore, placental pathology gives the most accurate estimate of an infant’s prenatal experience. The placenta is a unique vital organ that arises de novo and directly linked to the growth and development of the fetus in utero [11]. Its vital importance in the continuation of pregnancy and fetal nutrition, coupled with a scarcity of literature related to it, has led to great interest among obstetricians and pathologists to understand the “unique biological status” of this complex organ in patients with pre-eclampsia/eclampsia post-natally [11]. Additionally, recent literature has provided evidence of a significant association between placental pathology and intrauterine growth restriction/pre-eclampsia [12]. The histological changes in pre-eclamptic/eclamptic placentas include infarcts, increased syncytial knots, hypovascularity of the villi, cytotrophoblastic proliferation, thickening of the trophoblastic membrane, obliterative enlarged endothelial cells in the fetal capillaries, and atherosis of the spiral arteries in the placental bed [13].

The important reasons for devising a scoring system for histopathologists are as follows:

1) It would allow for a unified reporting system, which would assist in assessing placental histological changes regardless of geographical boundaries.

2) It would provide a simple approach to the pathologist in arriving at a specific diagnosis.

3) It would assist pathologists regarding the reproducibility of the diagnosis, thereby decreasing inter and intraobserver variability.

4) It would help in providing a baseline system for assessing pregnancy-induced hypertensive changes in the placenta for further validation and to include other parameters if necessary.

5) This system could be extrapolated in devising a comprehensive system for assessing all other causes of maternal uteroplacental insufficiency, such as gestational diabetes mellitus and Rh incompatibility.

Therefore, the primary aim of our study is to devise and validate a scoring system for assessing the histopathological changes in the placenta for the most accurate diagnosis of pre-eclampsia and eclampsia. The secondary aim is to engage in a comparative analysis of the histomorphological changes in the placentas of patients diagnosed with pre-eclampsia and eclampsia, and those with a normal placenta (control group).

## Materials and methods

We conducted a prospective study from November 2012 to December 2014 in the Department of Pathology, with the collaboration of the Department of Obstetrics and Gynecology, at the PES Institute of Medical Sciences and Research, Kuppam, Andhra Pradesh, India after obtaining the institutional review board (IRB) approval. All pregnant females above 18 years of age who had been diagnosed with pre-eclampsia and eclampsia and admitted for delivery or follow-up were included in the study. All cases of chronic hypertension, gestational hypertension, and chronic hypertension with superimposed pre-eclampsia were excluded. Additionally, all pre-eclampsia patients with co-existing conditions were also excluded.

A total of 100 cases were included, with 50 cases and 50 controls. The control patients were healthy pregnant females above 18 years of age who had a full-term normal delivery. After delivery, the placenta specimens were grossly examined according to protocols described by Driscoll et al. [14] and Robboy et al. [15] and subsequently fixed in 10% buffered formalin. The placental morphology, which included dimensions, cord length, and distance from the rupture of membranes, was recorded. Full-thickness (maternal floor to the fetal surface) placental bits were processed, and they consisted of normal tissue and areas suspicious of infarction, calcification, and hemorrhage. The membranes were sampled using the Swiss roll technique. Histopathological findings were recorded and graded according to the objective scoring system as discussed in Table 1. The cases were divided as per clinical diagnosis into pre-eclampsia, eclampsia, and controls, which were healthy normal patients.

**Figure 1:**
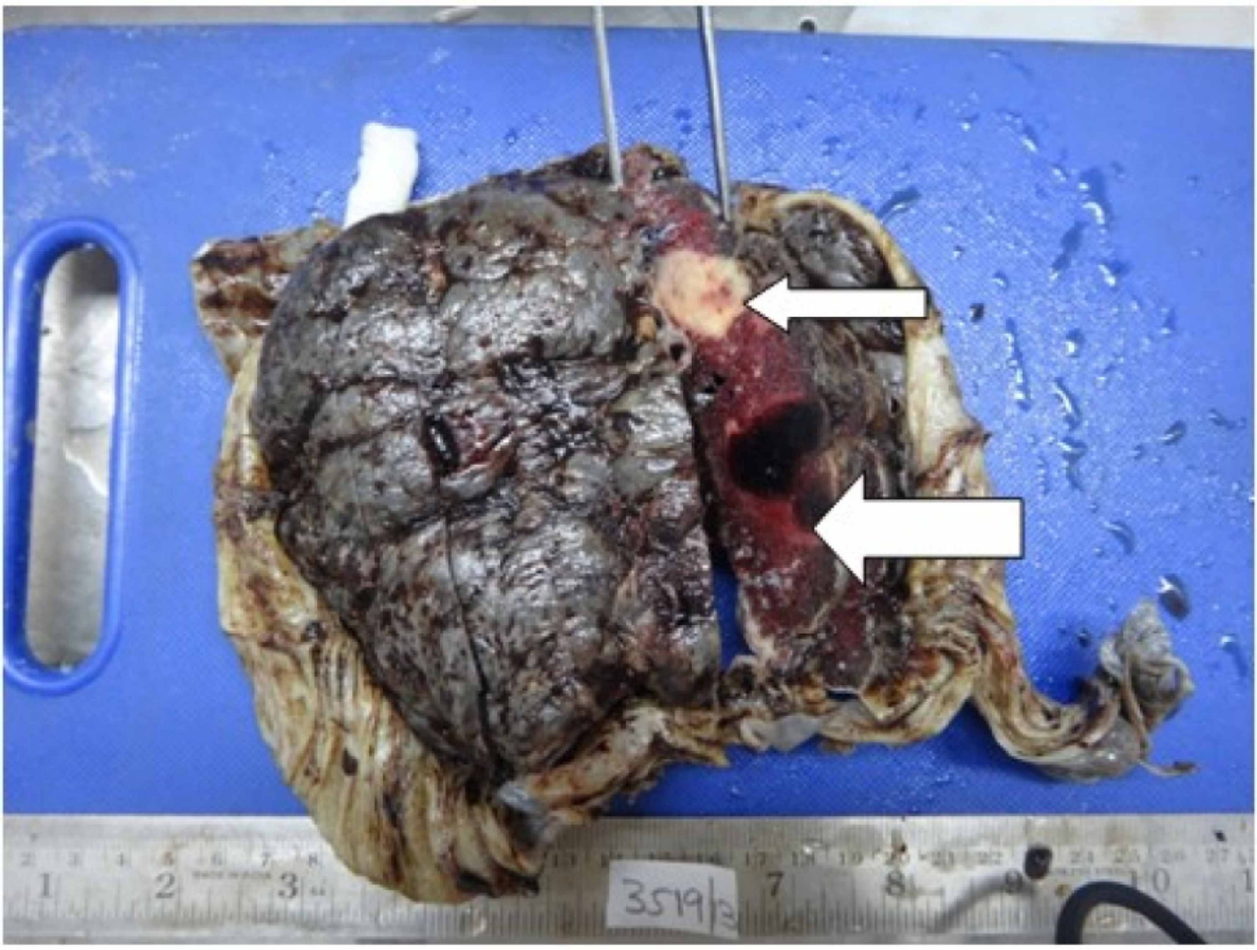
Examination of placenta specimen – image 1 The image shows infarction of placenta on gross examination Narrow arrow: white infarct; broad arrow: hemorrhagic infarct

**Figure 2:**
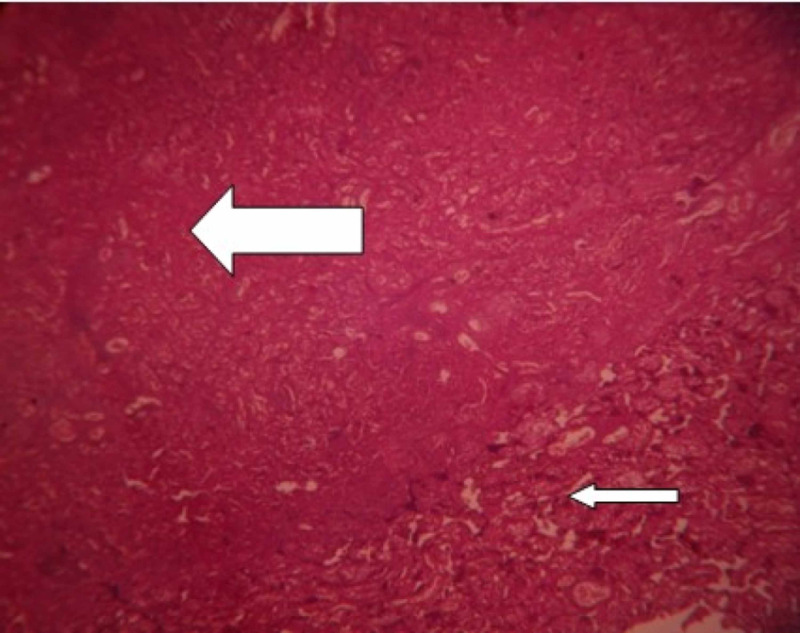
Examination of placenta specimen – image 2 Broad arrow: maternal floor infarction; narrow arrow: viable area (10X-H&E stain) H&E: hematoxylin and eosin

**Figure 3:**
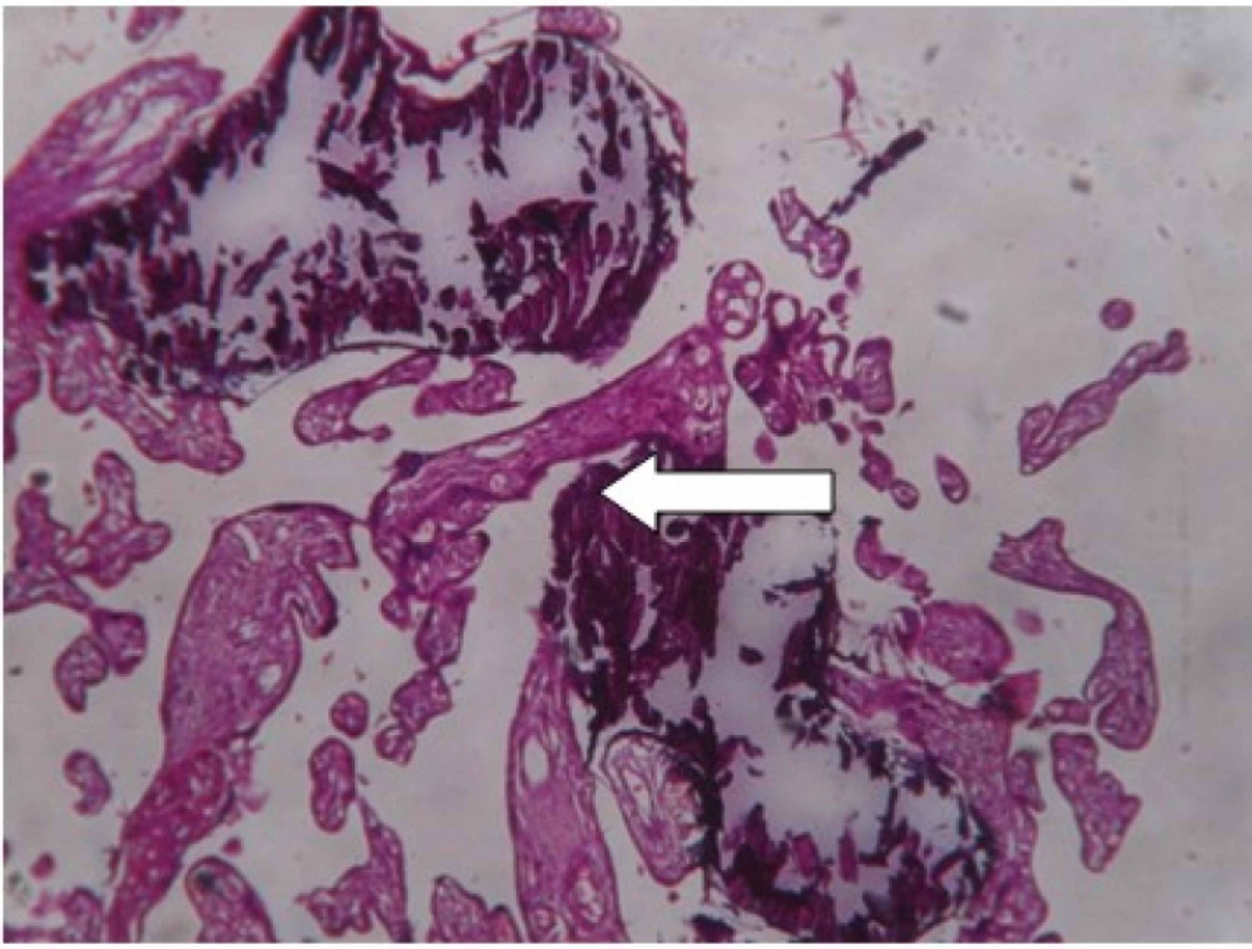
Examination of placenta specimen – image 3 The arrow shows calcification on microscopy (40X-H&E stain) H&E: hematoxylin and eosin

**Figure 4:**
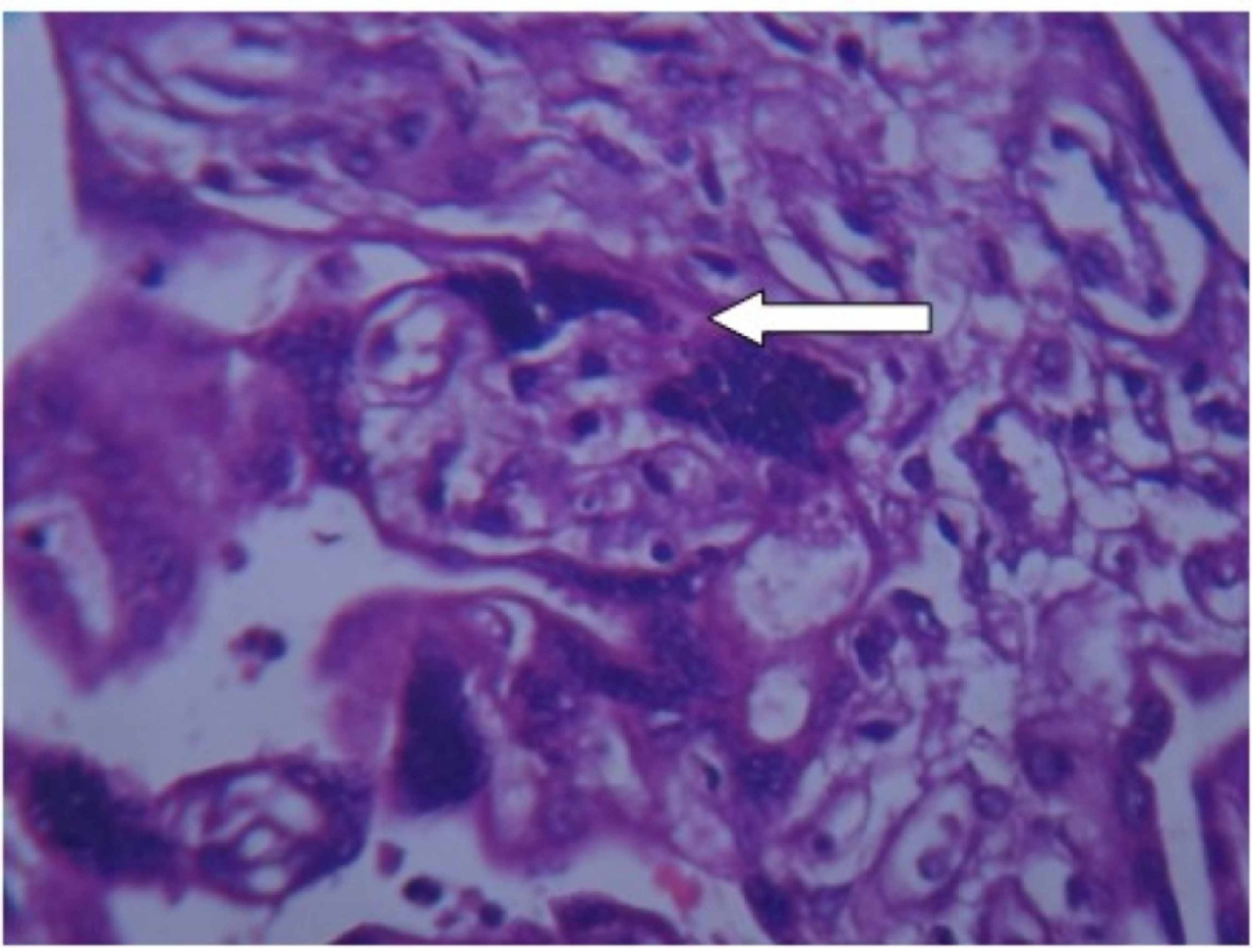
Examination of placenta specimen – image 4 The arrow shows syncytial knots on microscopy (40X-H&E stain) H&E: hematoxylin and eosin

**Figure 5:**
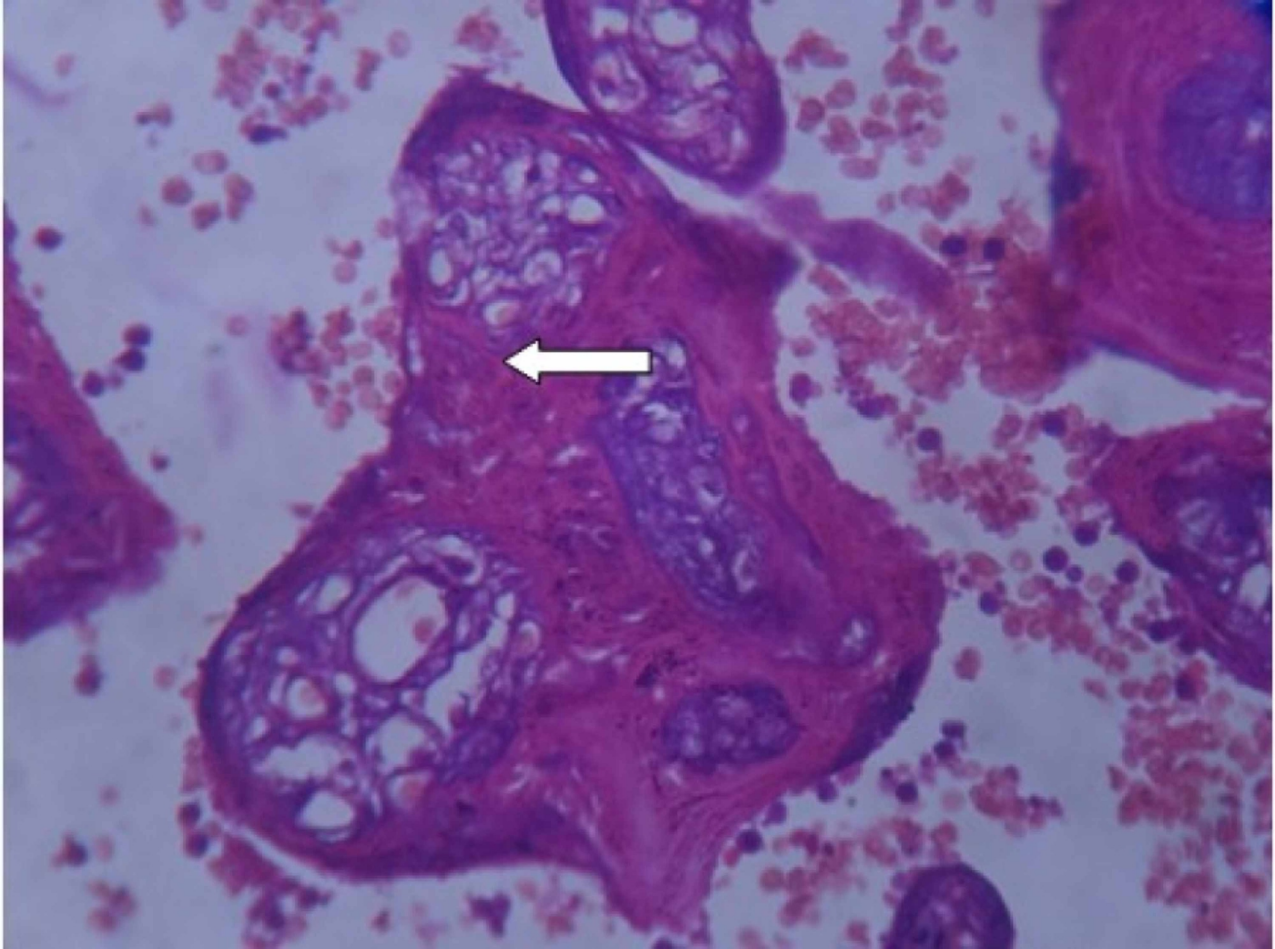
Examination of placenta specimen – image 5 The arrow shows perivillous fibrin deposit on microscopy (40X-H&E stain) H&E: hematoxylin and eosin

**Figure 6:**
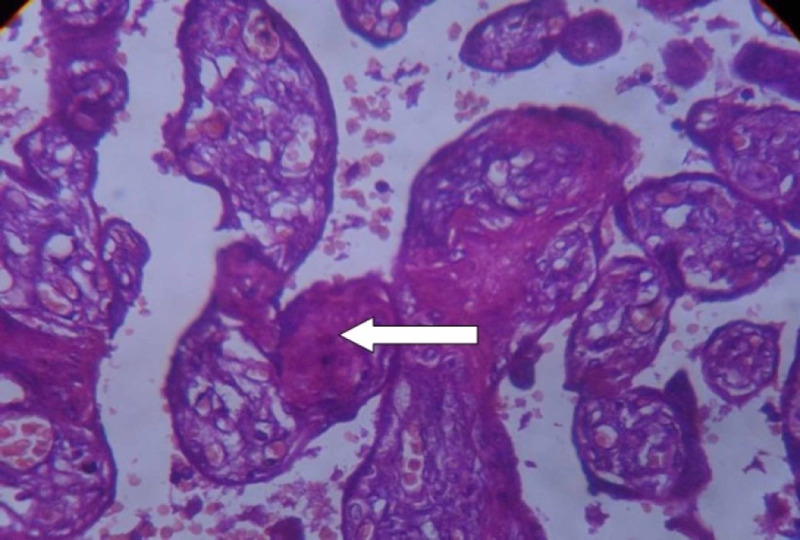
Examination of placenta specimen – image 6 The arrow shows intravillous fibrin deposit on microscopy (40X-H&E stain) H&E: hematoxylin and eosin

**Figure 7:**
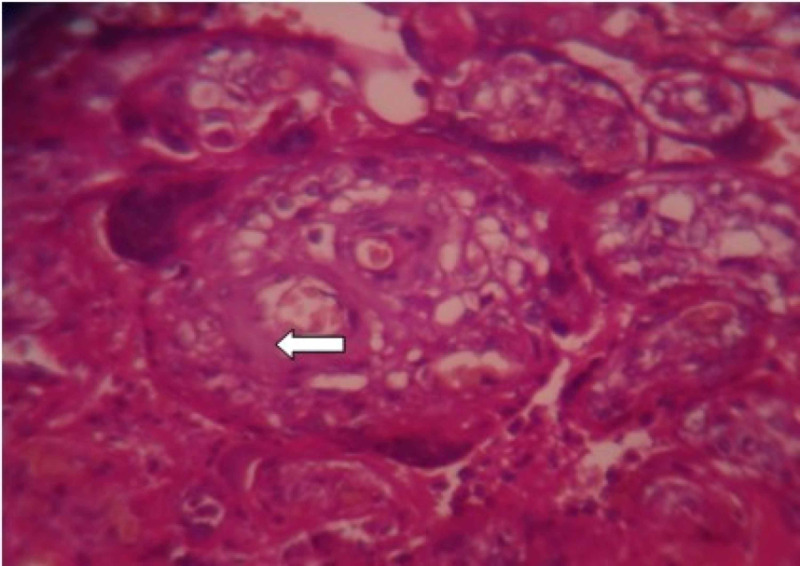
Examination of placenta specimen – image 7 The arrow shows villous basement membrane thickening on microscopy (40X-H&E stain) H&E: hematoxylin and eosin

We included five important histopathological parameters in the scoring system, namely fibrin deposition, maternal floor infarction, syncytial knots, calcification, and villous basement membrane thickening (Figures 1, 2, 3, 4, 5, 6, 7). Each parameter was given a minimum score of 0 and a maximum of 2, except for syncytial knots, which had a maximum score of 1 (since only histological presence or absence was considered). The scoring pattern for histopathological parameters is discussed in Table 1. The scores for these parameters were added up to provide a comprehensive score of a maximum of 9 and a minimum of 0.

**Table 1:** Scoring pattern for histopathological parameters considered in the analysis of pre-eclampsia and eclampsia placenta

Score/parameter	Fibrin deposition	Maternal floor infarction	Syncytial knots	Calcification	Villous basement membrane thickening
0	Histological absence	Histological absence, <5% of the infarcted area	Histological absence	Histological absence	Histological absence or <3% shows thickening
1	About 10% intravillous or perivillous fibrin	>5% of infarction in a single area	Histological presence	Seen in one lower field of a single section regardless of the number	>3% shows thickening
2	About 30% or more of both intravillous and perivillous fibrin deposits	>5% present in more than one area	-	Seen in multiple low power fields and also in multiple sections	Hyalinization changes

The analysis was done using SAS statistical software version 9.4 (SAS Institute, Cary, NC). Mean and standard deviations were used to describe the continuous variables. Categorical variables were represented using frequency and percentages. The association of various placental histopathological variables with a clinical diagnosis of pre-eclampsia, eclampsia, and control was analyzed using the chi-squared/Fisher’s exact test. The analysis of variance (ANOVA) test was used for comparing objective histopathological scores between pre-eclampsia, eclampsia, and control groups. A p-value of less than 0.05 was considered to be statistically significant.

## Results

We found that eclampsia patients had the highest prevalence of both intravillous and perivillous fibrin deposition compared to pre-eclampsia and control group respectively (40% vs. 17% vs. 2%; p<0.0001). Multiple areas of maternal floor infarction were more frequent in eclampsia compared to control and pre-eclampsia respectively (30% vs. 12% vs. 7%; p<0.0003). Eclampsia patients had 45% diffuse calcification compared to 33% in pre-eclampsia and 14% in the control group (p=0.018). The majority of the pre-eclampsia, eclampsia, and control group patients had normal villous basement membrane thickening. However, 15% of eclampsia patients had hyalinized villous basement membrane thickening compared to 0% in pre-eclampsia and control group patients (p<0.0001). Syncytial knots were more commonly present in eclampsia patients (75%) compared pre-eclampsia (73%) and control (26%) (p<0.0001) groups. Table 2 shows the distribution of various placental histomorphologic features in pre-eclampsia, eclampsia, and control group patients.

**Table 2:** Distribution of placental histomorphological features among pre-eclampsia, eclampsia, and control groups

	Pre-eclampsia	Eclampsia	Control	P-value
	Fibrin deposit			<0.0001
Intravillous/perivillous	23 (77%)	10 (50%)	18 (36%)	
Intravillous + perivillous	5 (17%)	8 (40%)	1 (2%)	
Absent	2 (7%)	2 (10%)	31 (62%)	
	Maternal floor infarction			0.0003
Single area	19 (63%)	11 (55%)	13 (26%)	
Multiple areas	2 (7%)	6 (30%)	6 (12%)	
Absent	9 (30%)	3 (15%)	31 (62%)	
	Calcification			0.018
Focal	13 (43.4%)	7 (35%)	18 (36%)	
Diffuse	10 (33.3%)	9 (45%)	7 (14%)	
Absent	7 (23.3%)	4 (20%)	25 (50%)	
	Villous basement membrane thickening			<0.0001
Normal	27 (90%)	8 (40%)	50 (100%)	
Thickened	3 (10%)	9 (45%)	0 (0%)	
Hyalinized	0 (0%)	3 (15%)	0 (0%)	
	Syncytial knots			<0.0001
Present	22 (73.3%)	15 (75%)	13 (26%)	
Absent	8 (27%)	5 (25%)	37 (74%)	

**Figure 8:**
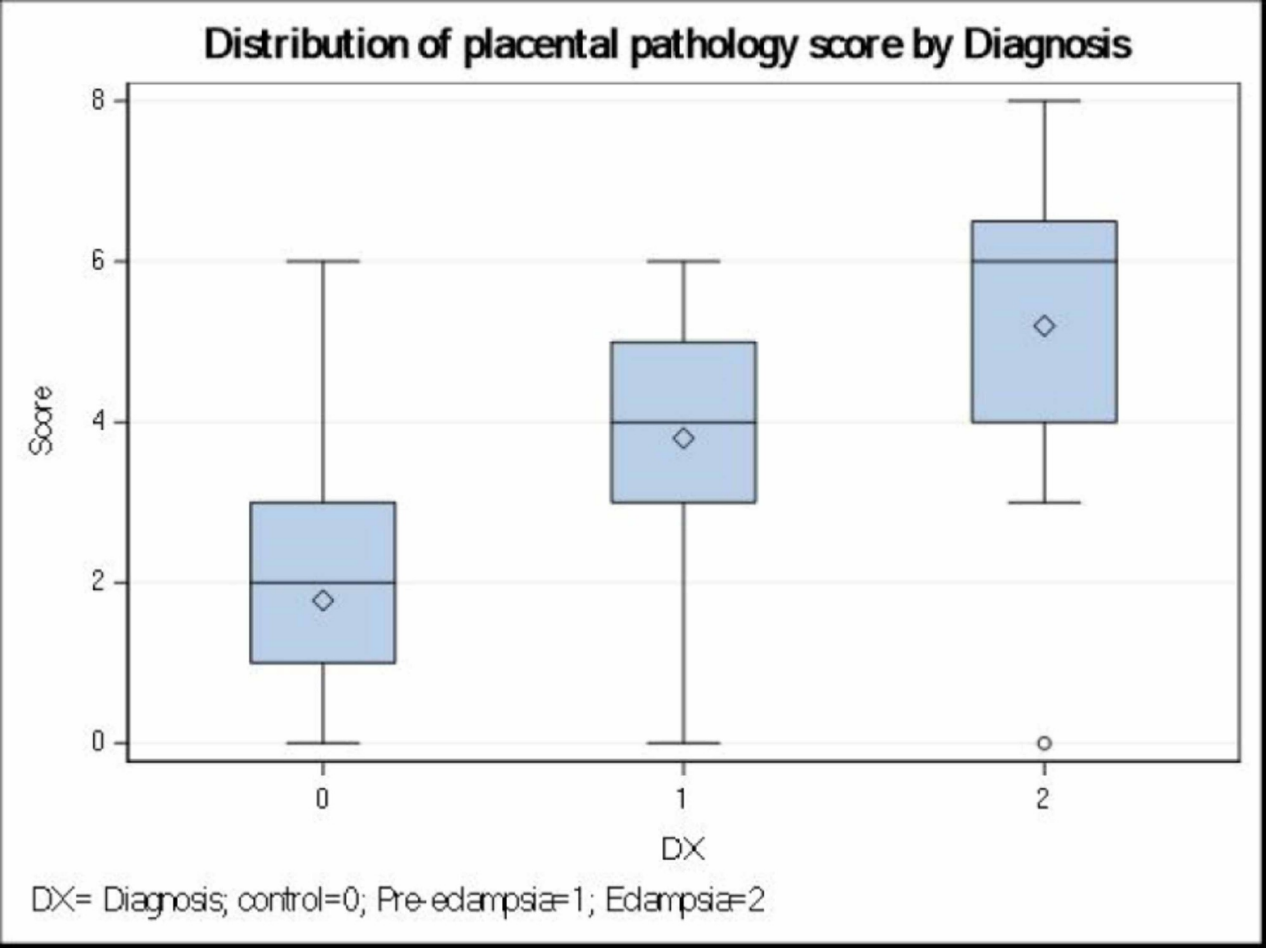
Placental pathology score by diagnosis

Additionally, we found a significant association between comprehensive histopathological scores of pre-eclamptic, eclamptic, and control groups with their clinical diagnosis (p<0.0001). A comprehensive median objective scores of 2, 4, and 6 were obtained for normal, pre-eclampsia, and eclampsia respectively (Table 3; Figure 8). These scores were significantly higher in eclamptic patients when compared to pre-eclamptic patients. The results suggest that the parameters considered for scoring the placental pathology showed significant histopathological differences between the clinically diagnosed pre-eclamptic and eclamptic patients.

**Table 3:** Association of comprehensive histopathological scores with clinical diagnosis IQR: interquartile range

Comprehensive histopathological Score	Pre-eclampsia	Eclampsia	Control	P-value
Median (IQR)	4 (2)	6 (2.5)	2 (2)	<0.0001

## Discussion

Our present study results showed a significant association between the objective histopathological scoring system, which consisted of fibrin deposition, maternal floor infarction, syncytial knots, calcification, and villous basement membrane thickening, and clinical diagnosis of pre-eclampsia and eclampsia. Additionally, we formulated a multi-parametric scoring system of placental pathology, which will be helpful in the diagnosis of pre-eclampsia and eclampsia. The placenta has been described as the mirror of perinatal mortality, and published literature reveals that pre-eclampsia/eclampsia syndrome has a deleterious effect on the placenta.

Fibrin entraps villi, obliterating the intervillous space and an important parameter that affects the villi and intervillous space [16]. When the syncytiotrophoblast of entrapped villi degenerates and disappears, persistent villous cytotrophoblast proliferates to form cellular mantle around individual villi. Extensive fibrin deposition involving more than 10% of the villous parenchyma is associated with fetal hypoxia and intrauterine growth retardation. Extensive infarcts in a background of markedly abnormal maternal vasculature and restricted maternal blood flow to the placenta underlie uteroplacental insufficiency of pre-eclampsia and eclampsia [17]. A study by Redline et al. [16] showed intervillous fibrin having the strongest correlation with both placental weight and fetal weight, which are in turn indicators for assessing maternal uteroplacental insufficiency [16]. These findings are similar to our findings where fibrin deposition was significantly associated with pre-eclampsia/eclampsia syndrome compared to controls. However, there is significant subjectivity in the assessment of such lesions as stated by Weber et.al. [18].

Maternal floor infarction involves fibrin deposition within or around the basal plate where basally situated chorionic villi are encased with fibrin [19]. Maternal floor infarction of more than 5% surface area has been considered pathological [20]. Our study findings of a higher frequency of maternal floor infarction in pre-eclampsia and eclampsia patients are consistent with the results of studies by Kambale et al. [20], Kurdukar et al. [21], and Ezeigwe et al. [22]. Furthermore, our findings of increased calcification found in pre-eclampsia and eclampsia patients are consistent with the findings of some other studies as well [20,21]. Also, the incidence of calcification increases as the severity of the hypertension increases. Dystrophic calcification of the placenta is evidence of placental senescence or degradation [22]. Calcium deposition noted in the villi and along the basement membrane strongly suggests uteroplacental insufficiency [23]. Syncytial nuclear aggregates are syncytiotrophoblast nuclei in clusters that can be found in sprouts, knots, and bridges. The number of knots is positively correlated with the length of time and severity of hypertensive diseases of pregnancy [24]. We found an increased frequency of the presence of syncytial knots in eclamptic (75%) and pre-eclamptic patients compared to the healthy control group. These findings are consistent with those of other studies [11,12,20,21]. It is speculated that hormonal factors could also influence syncytial knot formations in the placental villi, which leads to morphometrical changes in the placenta, resulting in pregnancy-induced hypertension [12]. However, the development of syncytial knots is not restricted to toxemia of pregnancy but also associated with excessive aging due to post-maturity or a disease state causing placental insufficiency [11].

Increased villi with thickened basement membrane occur due to ischemia of the uteroplacental circulation as seen in pre-eclampsia/eclampsia syndrome, which in turn is the result of the proliferation of cytotrophoblast and secretion of basement membrane protein. Hence, villous basement membrane thickening is secondary to placental ischemia consistent with pre-eclampsia [20]. Villous basement membrane thickening is considered to be present when more than 3% of the villous population of a section shows changes [11]. Interestingly, we found a higher prevalence of thickened basement membrane in eclamptic patients compared to pre-eclamptic and control groups, which is consistent with the findings of Kurdukar et al. and Narasimha et al. [11,21].

Strengths and limitations

One of the main limitations of our study was the small sample size. More accurate results could probably be deciphered with larger sample size. Secondly, our study did not assess the findings in relation to the fetal outcomes and focused only on changes in placenta parameters. Lastly, few confounding factors and undiagnosed subclinical conditions could have influenced maternofetal placental units causing changes, which could not be eliminated from the study. Despite all these limitations, the comprehensive histopathological scoring system in this study has significantly corroborated the severity of pre-eclampsia/eclampsia syndrome, viz., based on the scores obtained.

## Conclusions

We believe that the objective scoring system developed in this study can be a basis for validating reporting patterns of the placenta in pre-eclampsia and eclampsia patients. The study also elucidates a significant association between placental histopathological patterns, most likely due to maternal uteroplacental insufficiency, caused by pre-eclampsia and eclampsia. Hence, we feel we have laid the foundation for future, larger studies to validate the comprehensive histopathological scoring system not only towards diagnosing pre-eclampsia/eclampsia syndrome but also other similar diseases that cause maternal uteroplacental insufficiency. This will provide an efficient uniform reporting system for maternal uteroplacental insufficiency diseases. Also, we recommend that future studies include more etiopathogenetic factors such as hematological and clinical parameters to better assess the utility of the scoring system.
